# Misclassification Bias in the Waist‐To‐Hip Ratio: Implications for Obesity‐Related Risk of Incident Type 2 Diabetes

**DOI:** 10.1155/ije/1407398

**Published:** 2026-06-10

**Authors:** Si Han Hou, Jiao Wang, Tai Hing Lam, Lin Yang, Wei Sen Zhang, Lin Xu

**Affiliations:** ^1^ School of Public Health, Sun Yat-sen University, Guangzhou, China, sysu.edu.cn; ^2^ Guangzhou Twelfth People’s Hospital, Guangzhou, China; ^3^ School of Public Health, the University of Hong Kong, Hong Kong SAR, China, hku.hk; ^4^ School of Nursing, the Hong Kong Polytechnic University, Hong Kong SAR, China, polyu.edu.hk; ^5^ Department of Applied Health Sciences, School of Health Sciences, College of Medicine and Health, University of Birmingham, Birmingham, UK, birmingham.ac.uk

**Keywords:** body fat distribution, misclassification, risk stratification, Type 2 diabetes mellitus, waist-to-hip ratio

## Abstract

**Aims:**

Waist‐to‐hip ratio (WHR) directly reflects body fat distribution compared to other obesity indicators such as body mass index (BMI), waist circumference (WC), and waist‐to‐height ratio (WHtR). However, WHR may misclassify Type 2 diabetes mellitus (T2DM) risk by failing to account for the individual contribution of WC and hip circumference (HC). The study aimed to evaluate the discriminatory ability of WHR for T2DM and to identify its misclassification bias.

**Materials and Methods:**

This prospective cohort study included 15,211 Chinese participants aged 50+ years without T2DM at baseline (2003–08), with follow‐up conducted from 2008 to 2015. The Cox proportional hazard regression model was used to examine associations between obesity indicators and T2DM. Discriminatory ability was evaluated using the C‐statistic, net reclassification index (NRI), and integrated discrimination improvement (IDI).

**Results:**

During a median follow‐up of 5.4 years, 2238 participants (14.71%) developed T2DM. Participants with large WC and large HC had a higher T2DM risk compared to those with small WC and small HC in the low or high WHR category (adjusted hazard ratios (95% confidence intervals [CIs]): 2.33 [1.98, 2.74], 1.73 [1.47, 2.03]). WHR showed lower discrimination (C‐statistic 0.632, 95% CI 0.619, 0.645) than WC (0.645, 95% CI 0.632, 0.658) and WHtR (0.657, 95% CI 0.644, 0.670). Combining WHR with BMI significantly improved discrimination compared to WHR alone (△C‐statistic, NRI, IDI, and 95% CIs were 0.028 [0.020, 0.035], 0.015 [0.012, 0.020], and 0.141 [0.115, 0.165]), and it showed better discriminatory ability compared to the combination of BMI and WC (△C‐statistic 0.007, 95% CI 0.004, 0.011).

**Conclusions:**

WHR alone underestimates T2DM risk in individuals with large WC and large HC. Combining WHR with BMI addresses misclassification bias.

## 1. Introduction

Obesity is a well‐established and modifiable risk factor for both infectious and noninfectious diseases, particularly Type 2 diabetes mellitus (T2DM) [1], with abdominal adiposity playing a central role in its pathogenesis [2, 3]. Various anthropometric measures, such as body mass index (BMI), waist circumference (WC), waist‐to‐height ratio (WHtR), and waist‐to‐hip ratio (WHR), are widely used to assess obesity in clinical and epidemiological settings due to their practicality compared to direct measures of body fat [4]. Among these, WHR has garnered considerable attention as an indicator of body fat distribution, reflecting the balance between abdominal (upper‐body) and gluteofemoral (lower‐body) fat [5]. WHR has been linked to genetic variations in obesity independently of BMI [6] and is often cited as a recommended predictor of mortality [7] and cardiometabolic diseases [8–10].

Despite its widespread use, WHR is not without limitations. By focusing on the ratio of WC to hip circumference (HC), WHR may fail to account for the absolute values of these parameters. For example, individuals with large WC and large HC and those with small WC and small HC may have similar WHR values, despite potentially differing substantially in their overall risk profiles [11]. This lack of distinction introduces the potential for misclassification bias, which may undermine the discriminatory ability of WHR in identifying individuals at risk for T2DM. Furthermore, when WHR is dichotomized for risk stratification, it remains unclear whether individuals in the same WHR category but with differing absolute WC and HC measurements have comparable T2DM risk. This limitation has implications for both clinical risk assessment and the evaluation of obesity interventions, where changes in WHR may not accurately reflect changes in WC or HC [12].

To address these concerns, we conducted a prospective cohort study using data from the Guangzhou Biobank Cohort Study (GBCS). The study aimed to (1) evaluate the ability of WHR to discriminate the risk of incident T2DM compared to BMI, WC, and WHtR; (2) investigate the association between combinations of WC and HC categories and incident T2DM risk; and (3) explore approaches to address the misclassification bias of WHR in predicting incident T2DM risk. We hypothesized that individuals with large WC and large HC may have a significantly different risk of developing T2DM compared to those with small WC and small HC, even when categorized into the same WHR stratification. Furthermore, we proposed that combining WHR with BMI could enhance its discriminatory ability and reduce misclassification bias.

## 2. Materials and Methods

### 2.1. Study Population

GBCS is a three‐way collaborative prospective cohort study among the Guangzhou 12th People’s Hospital and the University of Hong Kong in China and the University of Birmingham in the UK. Details of GBCS have been reported elsewhere [13]. In brief, participants were recruited during 2003–2008 from Guangzhou Health and Happiness Association for the Respectable Elders (GHHARE), which was a community social and welfare organization including about 7% of Guangzhou residents in this age group. Membership was open to Guangzhou permanent residents aged 50 years or older for a nominal fee of 4 CNY (≈50 US cents) per month. Information on demographic characteristics, lifestyle factors, family, and personal medical history was collected at baseline by a face‐to‐face computer‐assisted questionnaire by trained nurses. Anthropometric measurements, blood pressure, fasting plasma glucose, and lipids were measured. Reliability and validity of the questionnaire were tested by recalling 200 randomly selected participants for re‐interview [13]. Ethics approval was granted by the Guangzhou Medical Ethics Committee of the Chinese Medical Association, and all participants provided written, informed consent before participation.

### 2.2. Obesity Indicators

Anthropometric measurements were measured by trained nurses using standard protocol in the morning before breakfast, encompassing weight, standing height, WC, and HC with light indoor clothing and without shoes. WC was measured horizontally around the smallest circumference between the ribs and iliac crest, or at the navel, if no natural waistline was present. HC was measured around the maximal girth of the hips. BMI was calculated as weight in kilograms divided by height in meters squared (kg/m^2^). WHtR was calculated by dividing WC in centimeters (cm) by height in cm, and WHR was calculated by dividing WC in cm by HC in cm. Detailed descriptions of the measurement procedures have been published elsewhere [13].

### 2.3. Outcomes

Fasting blood samples were obtained from all participants after an overnight fast, and glucose was determined automatically in the hospital laboratory. T2DM at baseline was defined as fasting glucose ≥ 7.0 mmol/L and/or self‐reported physician diagnosis of diabetes and/or antidiabetic treatment [14]. An oral glucose tolerance test (OGTT) was not done for those with a self‐reported physician diagnosis of diabetes or with antidiabetic treatment during the follow‐up. Otherwise, both fasting and 2‐h OGTT glucose were measured in all participants. Incident T2DM in those without T2DM at baseline was defined by fasting glucose ≥ 7.0 mmol/L, 2‐h OGTT ≥ 11.1 mmol/L, and/or self‐reported physician diagnosis of diabetes, and/or antidiabetic treatment during the follow‐up.

### 2.4. Potential Confounders

Potential confounders were considered in the analysis given the influence of factors such as sex, age, social‐economic characteristics (education, occupation, and family annual income) and lifestyles (smoking status, alcohol use, and physical activity) on both obesity and the development of T2DM [15, 16]. Education was classified into three levels: primary school or below, middle school, and college or above. Occupation was categorized as manual (agricultural work, factory work, or sales and services), nonmanual (administrative/managerial, professional/technical, or military/police), or others (housewife/husband or retired). Family annual income was grouped into five categories: < 10,000 CNY/year, 10,000–29,999 CNY/year, 30,000–49,999 CNY/year, ≥ 50,000 CNY/year, and unknown. Smoking status was categorized as never, former, and current smokers, and alcohol use was classified similarly as never, former, and current drinkers. Physical activity was assessed using the short version of the International Physical Activity Questionnaire (IPAQ), previously validated in our population, and categorized as inactive, moderate, or active [17].

### 2.5. Statistical Analysis

Differences of distribution in baseline characteristics by incident T2DM were compared using *t*‐test or Wilcoxon test for continuous variables and chi‐square test for categorical variables. The proportional hazard assumption was tested using the Schoenfeld residual method, and no violations were identified (all *p* > 0.05). Cox proportional hazard regression models, with follow‐up time as the time scale, were used to estimate the associations between various obesity indicators (i.e., BMI, WC, WHtR, and WHR) and the risk of incident T2DM. Additionally, the association between different combinations of WC and HC categories and T2DM risk was analyzed. For participants diagnosed with T2DM during follow‐up, the follow‐up time was defined as the interval from the baseline date to the midpoint between the baseline and follow‐up dates (1 March 2013–3 January 2020).

To allow for comparison of effect sizes among obesity indicators with different measurement units, each indicator was standardized into a sex‐specific z‐score using the formula: z‐score= (actual value − mean)/standard deviation (SD). The discriminatory ability of WHR for predicting incident T2DM was compared with other obesity indicators using several metrics: the C‐statistic, continuous net reclassification index (NRI), and integrated discrimination improvement (IDI). NRI and IDI were chosen as they are considered more sensitive and clinically relevant measures for evaluating improvements in discriminatory ability than the C‐statistic [18].

In the analyses of the associations of combinations of WHR, WC and HC categories with T2DM risk, these variables were divided into groups based on sex‐specific medians (WHR: women: 0.84, men: 0.90; WC: women: 76 cm, men: 81 cm; HC: women: 90 cm; men: 90 cm) rather than standard clinical cut‐offs, ensuring sufficient sample sizes within each category. The reference group comprised participants with small WC and small HC in the low WHR stratification. When exploring the stratified risks of incident T2DM after combining BMI and WHR, BMI was categorized according to the Chinese criteria (normal weight: < 24 kg / *m* ^2^, overweight: 24 kg / *m* ^2^ ≤ BMI < 28 kg / *m* ^2^; general obesity: ≥ 28 kg / *m* ^2^) [19]. Discriminatory ability was reassessed after combining BMI with central obesity indicators (i.e., WHR, WC, and WHtR) using the C‐statistic, NRI, and IDI.

Sensitivity analyses were conducted to assess the robustness of our findings. First, given the established sex differences in body fat distribution and its association with T2DM risk, all analyses were stratified by sex. Second, potential confounding by dietary factors and family history of diabetes were addressed by further adjusting models for daily total energy intake and family history of diabetes, respectively. Daily total energy intake was assessed by a validated food frequency questionnaire (FFQ) that collected data on the frequency and amount of food consumed over the past 7 days [20]. Third, considering the clinical generalizability of our results, we also used criteria from the World Health Organization (WHO) instead of using sex‐specific medians to classify WC and WHR (cut‐off value of WHR: women: 0.85, men: 0.90; cut‐off value of WC for Chinese: women: 80 cm, men: 90 cm) [21]. Stata/SE 17.0 (Stata Corp LP) and *R* software (Version 4.4.2) were used for data analyses. All tests were two‐sided with a significant level of *p* < 0.05.

## 3. Results

### 3.1. Baseline Characteristics

By 31 December 2020, vital status was determined for 30,097 participants in the study. After excluding 11,823 participants who did not complete follow‐up, 18,274 participants remained. Of them, 953 participants were excluded due to missing data on body measurement indicators, T2DM status at baseline or follow‐up, or confounding factors, and an additional 2110 participants with T2DM at baseline were excluded. Thus, a total of 15,211 participants were included in the main analyses (Figure S1). During a median follow‐up period of 5.4 years, 2238 participants (14.71%) were newly diagnosed with T2DM, including 1638 women (73.19%) and 600 men (26.81%).

Table 1 shows that participants who developed T2DM were older, more likely to have lower levels of education and have nonmanual work, report former alcohol use, and have lower family annual income (all *p* < 0.05). No significant differences were observed for sex or smoking status, or physical activity. Anthropometric and obesity indicators differed significantly between the groups, with participants who developed T2DM showing higher weight, WC, HC, BMI, WHtR, and WHR and shorter stature compared to those who did not develop T2DM (all *p* < 0.001).

**Table TABLE 1:** Baseline characteristics of participants by incident T2DM status.

	Non‐T2DM	Newly developed T2DM	*p* value
Number of participants, N (%)	12,973 (85.29)	2238 (14.71)	—
Age, years, mean (SD)	60.76 (6.87)	61.88 (6.47)	< 0.001
Sex, N (%)			0.683
Women	9441 (72.77)	1638 (73.19)	
Men	3532 (27.23)	600 (26.81)	
Education, N (%)			< 0.001
Primary school or below	4825 (37.19)	971 (43.39)	
Middle school	6970 (53.73)	1069 (47.77)	
College or above	1178 (9.08)	198 (8.85)	
Occupation, N (%)			0.024
Manual	7820 (60.28)	1353 (60.46)	
Nonmanual	3116 (24.02)	578 (25.83)	
Others	2037 (15.70)	307 (13.72)	
Family annual income, CNY/year, N (%)			0.023
< 10,000	64 (4.99)	102 (4.56)	
10,000–29,999	4089 (31.52)	739 (33.02)	
30,000–49.999	2997 (23.10)	464 (20.73)	
≥ 50,000	2396 (18.47)	395 (17.65)	
Don’t know	2844 (21.92)	538 (24.04)	
Smoking, N (%)			0.414
Never	1067 (82.25)	1830 (81.77)	
Former	1055 (8.13)	200 (8.94)	
Current	1248 (9.62)	208 (9.29)	
Alcohol use, N (%)			0.035
Never	9198 (70.90)	1629 (72.79)	
Former	43 (3.38)	87 (3.89)	
Current	3337 (25.72)	522 (23.32)	
Physical activity, N (%)			0.104
Inactive	1108 (8.54)	166 (7.42)	
Moderate	5047 (38.90)	910 (40.66)	
Active	6818 (52.56)	1162 (51.92)	
Anthropometric indicators			
Height, cm, mean (SD)	156.89 (7.28)	156.21 (7.45)	< 0.001
Weight, kg, mean (SD)	57.69 (9.31)	61.25 (9.73)	< 0.001
Waist circumference, cm, mean (SD)	77.31 (8.58)	82.28 (8.54)	< 0.001
Hip circumference, cm, mean (SD)	90.28 (6.12)	92.57 (6.55)	< 0.001
Obesity indicators			
Body mass index, kg / *m* ^2^, mean (SD)	23.40 (3.15)	25.02 (3.26)	< 0.001
Waist‐to‐height ratio, mean (SD)	0.49 (0.05)	0.53 (0.05)	< 0.001
Waist‐to‐hip ratio, mean (SD)	0.86 (0.07)	0.89 (0.06)	< 0.001

CNY = Chinese yuan, SD = standard deviation.

### 3.2. The Discriminatory Ability of WHR Compared to Other Obesity Indicators

In Table 2, after adjusting for confounding factors, the strength of association with incident T2DM was greater for WC and WHtR than for WHR and BMI. The HRs (95% CIs) for each SD increment were 1.62 (1.56, 1.69) for WC, 1.67 (1.60, 1.74) for WHtR, 1.50 (1.45, 1.56) for WHR, and 1.55 (1.49, 1.61) for BMI. In terms of discriminatory ability, WHR and BMI showed comparable C‐statistics (WHR: 0.626 (95% CI 0.615, 0.637); BMI: 0.635 (95% CI 0.624, 0.646)), with no significant differences in C‐statistic (△C‐statistic 0.008, 95% CI ‐0.004, 0.021). WHtR showed a higher C‐statistic than WHR, with differences in △C‐statistic, NRI, and IDI reported as 0.027 (95% CI 0.018, 0.037), 0.110 (95% CI 0.079, 0.145), and 0.015 (95% CI 0.011, 0.020), respectively. WC showed similar results, with differences in △C‐statistic, NRI, IDI compared to WHR being 0.016 (95% CI 0.007, 0.024), 0.087 (95% CI 0.048, 0.128), and 0.009 (95% CI 0.005, 0.013), respectively.

**Table TABLE 2:** Comparisons of obesity indicators for associations with risk of incident T2DM and their discriminatory performance.

	HR (95% CI)^†^	C‐statistic (95% CI)	ΔC‐statistic (95% CI)	NRI (95% CI)	IDI (95% CI)
BMI (1 SD = 3.26 kg/m^2^ in women; 3.10 kg/m^2^ in men)	1.55 (1.49, 1.61)^∗∗∗^	0.635 (0.624, 0.646)	0.008 (−0.004, 0.021)	0.024 (−0.015, 0.064)	0.006 (0.000, 0.012)^∗^
WC (1 SD = 8.36 cm in women; 8.86 cm in men)	1.62 (1.56, 1.69)^∗∗∗^	0.642 (0.631, 0.653)	0.016 (0.007, 0.024)^∗∗∗^	0.087 (0.048, 0.128)^∗∗∗^	0.009 (0.005, 0.013)^∗∗∗^
WHtR (1 SD = 0.06 in women; 0.05 in men)	1.67 (1.60, 1.74)^∗∗∗^	0.654 (0.643, 0.665)	0.027 (0.018, 0.037)^∗∗∗^	0.110 (0.079, 0.145)^∗∗∗^	0.015 (0.011, 0.020)^∗∗∗^
WHR (1 SD = 0.06 in women; 0.06 in men)	1.50 (1.45, 1.56)^∗∗∗^	0.626 (0.615, 0.637)	0.000 (ref)	0.000 (ref)	0.000 (ref)

SD = standard deviation, HR = hazard ratio, CI = confidence interval, NRI = net reclassification index, IDI = integrated discrimination improvement, BMI = body mass index, WHtR = waist‐to‐height ratio, WHR = waist‐to‐hip ratio, WC = waist circumference, HC = hip circumference.

^†^Adjusted for age, sex, education, occupation, family income, smoking status, alcohol use, physical activity.

^∗^ *p* < 0.05.

^∗∗∗^ *p* < 0.001.

### 3.3. Associations of WC and HC With Incident T2DM in Separate WHR Stratification

Within both the low and high WHR groups, WC was positively associated with incident T2DM (Table S1). This association was stronger after adjusting for HC. In the low WHR group, the HRs (95% CIs) for each SD increment in WC were 1.80 (1.64, 1.98) before and 2.80 (2.18, 3.61) after adjustment for HC. Similarly, in the high WHR group, the HRs (95% CIs) were 1.44 (1.35, 1.53) before and 1.54 (1.40, 1.71) after adjustment for HC (Table S1). In the low WHR group, HC had an inversely negative association with incident T2DM when adjusting for WC (HR: 0.71, 95% CI 0.59, 0.85).

Table 3 shows that in the low WHR group, participants with large WC and large HC had a 133% higher risk of incident T2DM compared to those with small WC and small HC (HR 2.33, 95% CI 1.98, 2.74). In the high WHR group, the category with large WC and large HC also showed a higher risk of incident T2DM compared to the category with small WC and small HC (HR 1.73, 95% CI 1.47, 2.03).

**Table TABLE 3:** Associations of WC and HC categories with risk of incident T2DM across WHR stratifications.

	Newly developed T2DM/Non‐T2DM	Incidence rate, per 1000 Person‐years	HR (95% CI)^†^
Low WHR^a^			
Small WC and small HC^b^	291/3767	11.76	1.00 (ref)
Small WC and large HC	140/1534	13.58	1.18 (0.96, 1.44)
Large WC and small HC	—	—	—
Large WC and large HC	296/1495	28.18	2.33 (1.98, 2.74)^∗∗∗^
High WHR^a^			
Small WC and small HC^b^	171/1107	23.57	1.00 (ref)
Small WC and large HC	—	—	—
Large WC and small HC	266/1281	31.33	1.32 (1.08, 1.59)^∗∗^
Large WC and large HC	1074/3789	41.39	1.73 (1.47, 2.03)^∗∗∗^

WHR = waist‐to‐hip ratio, WC = waist circumference, HC = hip circumference, HR = hazard ratio, CI = confidence interval.

^a^WHR were divided by the sex‐specific medians (women: 0.84; men: 0.90).

^b^Small and large WC were divided by the sex‐specific medians (women: 76 cm; men: 81 cm). Small and large HC were divided by the sex‐specific medians (women: 90 cm; men: 90 cm).

^†^Adjusted for age, sex, education, occupation, family income, smoking status, alcohol use, physical activity.

^∗∗^ *p* < 0.01.

^∗∗∗^ *p* < 0.001.

### 3.4. Combining WHR and BMI on Incident T2DM Discrimination

Significantly positive correlations were observed between BMI and both WC and HC (Pearson’s *r* = 0.77 and 0.78, respectively, both *p* < 0.001; Figure S2). Additionally, participants with large WC and large HC showed significantly higher BMI and a higher prevalence of overweight and general obesity compared to those with small WC and small HC (Figure S3, Figure S4). Based on these findings, BMI was incorporated alongside WHR to better distinguish participants with large WC and large HC from those with small WC and small HC and to address potential misclassification bias of T2DM risk by WHR.

In the low WHR group, participants with large WC and large HC combined with overweight and general obesity had a significantly higher risk of incident T2DM compared to those with small WC and small HC combined with normal weight (HR 2.44, 95% CI 2.02, 2.95, and 3.18, 95% CI 2.36, 4.29, respectively; Figure 1A, Table S2). In the high WHR group, similar associations were observed. The HRs (95% CIs) for participants with large WC and large HC combined with overweight and general obesity were 1.58 (1.32, 1.89) and 2.68 (2.22, 3.24), respectively, compared to those with small WC and small HC combined with normal weight (Figure 1A, Table S2).

**Figure FIGURE 1:**
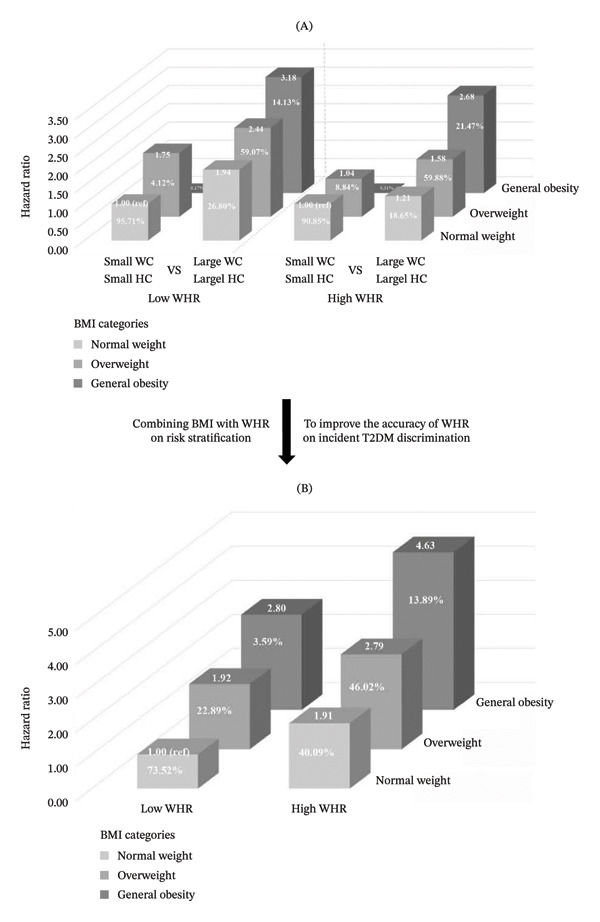
Associations of WC and HC categories combined with BMI on incident T2DM risk across WHR stratifications. Note: BMI was classified according to the Chinese criteria (normal weight: < 24 kg / *m* ^2^, overweight: 24 kg / *m* ^2^ ≤ BMI< 28 kg / *m* ^2^; general obesity: ≥ 28 kg / *m* ^2^). Low and high WHR were divided by the sex‐specific medians (women: 0.84; men: 0.90). Small and large WC were divided by the sex‐specific medians (female: 76 cm; male: 81 cm). Small and large HC were divided by the sex‐specific medians (women: 90 cm; men: 90 cm). T2DM = Type 2 diabetes, BMI = body mass index, WHR = waist‐to‐hip ratio, WC = waist circumference, HC = hip circumference.

The joint associations of WHR and BMI with incident T2DM risk are shown in Figure 1B and Table S3. In the low WHR group, overweight and general obesity were associated with higher T2DM risk compared to normal weight, with HRs (95% CIs) of 1.92 (1.64, 2.25) and 2.80 (2.10, 3.72), respectively. Similarly, in the high WHR group, HRs (95% CIs) for overweight and general obesity were 2.79 (2.47, 3.15) and 4.63 (4.01, 5.35), respectively.

Table 4 shows the discriminatory ability of combining BMI with central obesity indicators. The C‐statistic increased from 0.626 (95% CI 0.615, 0.637) for WHR alone to 0.654 (95% CI 0.643, 0.665) when combining BMI and WHR (△C‐statistic 0.028, 95% CI 0.020, 0.035). The combination of BMI and WHR also showed a higher NRI and IDI compared to WHR alone (NRI 0.015, 95% CI 0.012, 0.020; IDI 0.141, 95% CI 0.115, 0.165). Compared to the combination of BMI with WC, the combinations of BMI with WHR provided better discriminatory ability (△C‐statistic −0.007, 95% CI ‐0.011, −0.004; NRI ‐0.107, 95% CI ‐0.142, −0.070; IDI ‐0.003, 95% CI ‐0.005, −0.001). Combining BMI with WHR or WHtR showed comparable discriminatory ability (△C‐statistic 0.000, 95% CI ‐0.004, 0.005; NRI ‐0.019, 95% CI ‐0.047, 0.023; IDI 0.001, 95% CI ‐0.001, 0.004).

**Table TABLE 4:** Comparisons of discriminatory performance of combined BMI and central obesity indicators for predicting incident T2DM.

	C‐statistic (95% CI)	ΔC‐statistic (95% CI)	NRI (95% CI)	IDI (95% CI)
BMI + WC	0.647 (0.636, 0.658)	−0.007 (−0.011, −0.004)^∗∗∗^	−0.107 (−0.142, −0.070)^∗∗∗^	−0.003 (−0.005, −0.001)^∗∗∗^
BMI + WHtR	0.655 (0.644, 0.666)	0.000 (−0.004, 0.005)	−0.019 (−0.047, 0.023)	0.001 (−0.001, 0.004)
BMI + WHR	0.654 (0.643, 0.665)	0.000 (ref)	0.000 (ref)	0.000 (ref)
WHR alone	0.626 (0.615, 0.637)	−0.028 (−0.035, −0.020)^∗∗∗^	−0.015 (−0.020, −0.012)^∗∗∗^	−0.141 (−0.165, −0.115)^∗∗∗^

CI = confidence interval, NRI = net reclassification index, IDI = integrated discrimination improvement, BMI = body mass index, WC = waist circumference, WHtR = waist‐to‐height ratio, WHR = waist‐to‐hip ratio.

^∗∗∗^ *p* < 0.001.

### 3.5. Sensitivity Analysis

Figure S4 shows significant differences in incident T2DM risk between participants with small WC and small HC and those with large WC and large HC with the same WHR stratification, in both women and men. In women, WHR showed inferior performance compared to other measures (Table S4). Similar to the main results, the discriminatory ability of WHR improved after combining with BMI (Table S5). In men, significant improvement in discriminatory ability was observed when WHR was combined with BMI compared to WHR alone (Table S4, Table S5). This misclassification of incident T2DM risk by WHR was diminished when BMI was included in the risk stratification (Table S6, Table S7). When further adjusting for daily energy intake (*n* = 9941) or family history of diabetes (*n* = 4993), distinct differences in T2DM risk persisted between participants with small WC and small HC and those with large WC and large HC (Table S8, Table S11). However, incorporating BMI into the classification effectively distinguished the T2DM risk of participants with large WC and large HC (Table S9‐S10, Table S12‐S13). When using WHO criteria to classify WHR and WC, we also found that combing WHR and BMI could reduce the misclassification bias of WHR alone (Table S14‐S16).

## 4. Discussion

This study highlights the limitations of WHR as a standalone measure of obesity in predicting the risk of incident T2DM. While WHR is a widely used indicator of fat distribution, our findings demonstrate significant misclassification of T2DM risk within the same WHR stratification, particularly for individuals with large WC and large HC versus those with small WC and small HC. This misclassification was evident across both sexes and persisted after adjustment for potential confounders. However, the addition of BMI to WHR improved risk discrimination, reducing the observed misclassification and enhancing the ability to stratify T2DM risk effectively. These results underscore the need to consider complementary obesity indicators, such as BMI alongside WHR, to improve the assessment of obesity‐related T2DM risk in both clinical and epidemiological contexts.

WHR is an obesity indicator that captures the characteristics of both WC and HC, reflecting body fat distribution. The inclusion of both measurements in WHR is theoretically based on the distinct physiological effects of abdominal and gluteofemoral fat depots. Specifically, abdominal visceral fat is considered a primary driver of metabolic dysfunction, while gluteofemoral fat exhibits relatively protective properties [22]. Previous mechanistic studies observed that gluteofemoral subcutaneous adipose tissue exhibits lower lipolytic activity, which effectively curtails the release of nonesterified free fatty acids into the bloodstream [23]. Additionally, the gluteofemoral fat depot possesses a capacity to accommodate excess energy via adipocyte hyperplasia. This function is associated with a favorable profile of adipokines, characterized by elevated levels of adiponectin and diminished secretion of proinflammatory cytokines, which collectively preserve insulin sensitivity [24]. In recent years, several novel obesity indicators such as a body shape index (ABSI) [25] and body roundness index (BRI) [26] have been developed. Although these indicators provide improved mathematical modeling of abdominal adiposity, they often focus primarily on the detrimental effects of the abdominal visceral fat depot without explicitly incorporating the independent metabolic contribution of the gluteofemoral fat depot.

Despite the theoretical strength of WHR in integrating these two metrics, the reliance on a ratio‐based format introduces an interpretative limitation. WHR cannot distinguish between individuals who have high absolute values for both WC and HC and those who have low values for both, provided their proportions are similar. This mathematical neutralization may overlook the overwhelming pathological effect of absolute abdominal visceral adiposity. Our findings provide epidemiological evidence for this misclassification bias. We found that participants with large WC and large HC had a significantly higher risk of incident T2DM compared with those with small WC and small HC, despite being categorized with the same WHR stratification. These results suggest that the observed higher risk is primarily attributable to large WC. This observation also provides insight into why WHR showed weaker discriminatory ability compared with WC and WHtR, despite being the only obesity indicator that integrates WC and HC. Our findings highlight the importance of carefully considering the applicability of WHR in health education and public health practice. For example, WHR is often used to classify individuals as having a “healthy” pear‐shaped body (WHR below a cut‐off) or an “unhealthy” apple‐shaped body (WHR above a cut‐off) [27]. However, a “healthy” body shape indicated by WHR may not necessarily reflect small WC, and attention should still be given to individuals with large WC who may be at increased risk of T2DM and other metabolic diseases despite being categorized as pear‐shaped.

To address this limitation, we further proposed combining WHR with BMI to enhance the discriminatory ability of WHR for incident T2DM. Although BMI does not measure fat distribution or distinguish between muscle and fat mass [28], it can differentiate individuals with small WC and small HC from those with large WC and large HC. Our analyses showed that the combination of BMI and WHR effectively discriminated the incident T2DM risk associated with large WC and large HC. Individuals with overweight and general obesity had higher T2DM risk compared to those with normal weight, regardless of whether they were in the low or high WHR group, consistent with previous studies [29, 30]. Furthermore, combining BMI with WHR showed better discriminatory performance compared with the combinations of BMI and other central obesity indicators, including WC. The effectiveness of combining BMI and WHR may vary by ethnicity and study design. A Swedish cohort study among adults aged 45–73 with a 14‐year follow‐up found that adding BMI to WHR improved discrimination compared with WHR alone, but no significant differences were observed between the combination of BMI and WHR and the combinations of BMI and other central obesity indicators [31]. Conversely, a cross‐sectional study among Cameroon adults without diagnosed T2DM found that the combination of BMI and WHR did not enhance discriminatory ability compared with WHR alone and showed no improvement compared with the combination of BMI and WC [32].

Current clinical consensus has also increasingly emphasized the necessity of integrating BMI with other measurements of abdominal obesity to more accurately assess obesity and predict metabolic risk [33, 34]. This comprehensive assessment strategy is particularly essential in the Asian population due to the ethnic‐specific body fat distribution phenotype. The Asian population exhibits a higher body fat percentage and a more pronounced abdominal fat distribution at the same BMI range compared to the white population [35]. Therefore, the combination of BMI and abdominal obesity indicators is not merely a statistical refinement but a mechanistic necessity to better differentiate the divergent metabolic profiles among Asian individuals. However, the optimal abdominal obesity indicator combined with BMI remains inconclusive, as different obesity indicators may vary in predictive performance across diverse populations.

In this study, WHR showed weaker discriminatory ability for predicting incident T2DM compared to WC and WHtR and performed similarly to BMI when evaluated using the C‐statistic, NRI, and IDI. These findings align with a UK Biobank study involving 161,127 white European participants, which reported that over a median follow‐up of 10 years, WHtR and BMI had significantly higher C‐statistics than WHR [36]. Conversely, data from the Atherosclerosis Risk in Communities study, which included 12,121 White and Black adults followed for more than 11 years, showed that BMI, WC, WHtR, and WHR all served as comparable discriminators of T2DM [37]. A study from the Taiwan Biobank, including 24,346 participants with a median 4‐year follow‐up, suggested sex differences in the discrimination of obesity indicators for T2DM [38]. In this study, the area under curve (AUC) for WHtR and WHR was comparable in men, whereas WHtR had higher AUC than WHR in women [38]. While these prospective studies provide valuable insights, inconsistencies in their findings highlight a lack of focus on the underlying reasons for the varying discrimination ability of obesity indicators. This gap may indicate the development of improved measures for assessing obesity.

This study has several strengths, including the high precision of anthropometric measurements conducted by well‐trained nurses and the comprehensive assessment of the discriminatory ability of various obesity indicators using C‐statistic, NRI, and IDI. Most notable, this is the first study to identify and quantify the misclassification bias of WHR in assessing T2DM risk. We showed that participants with large WC and large HC are at significantly higher risk of incident T2DM compared to those with small WC and small HC, despite being categorized into the same WHR stratification. However, this study also had some limitations. First, the reliance on baseline anthropometric data does not account for changes in obesity status over time. Further studies using repeated measurements would provide a more accurate estimation. Second, as all participants were adults aged 50 years and older from southern China, caution is warranted when extrapolating these findings to younger populations or other ethnic groups. Further studies are needed to validate the discrimination of WHR alone and in combination with BMI in diverse populations. Third, residual confounders might exist, as factors such as family history of obesity were not available. However, given that our participants were predominantly born between the 1930s and 1950s, their parents lived during periods of substantial socioeconomic deprivation in China when obesity was relatively uncommon, potentially limiting the relevance of this variable in our study population. Additionally, the sensitivity analysis adjusting for family history of diabetes, one of the key indicators of predisposition to metabolic disorders, showed consistent results.

## 5. Conclusions

Overall, this study is the first to highlight the misclassification bias in WHR, showing its inability to fully capture the increased T2DM risk of individuals with large WC and large HC. Although this limitation constrains WHR’s application as a standalone measure, combining WHR with BMI significantly improves its discriminatory performance.

## Funding

The research and publication of their article was supported by the National Natural Science Foundation of China (grant numbers 82373661).

## Conflicts of Interest

The authors declare no conflicts of interest.

## Supporting Information

Additional supporting information can be found online in the Supporting Information section.

## Supporting information


**Supporting Information** The supporting information file includes the flowchart of the study participant, supporting tables and figures of the main analysis, and the results of sensitivity analysis.

## Data Availability

Due to ethical restrictions protecting patient privacy, data are available on request from the Guangzhou Biobank Cohort Study Data Access Committee. Please contact us at gbcsdata@hku.hk for the study protocol, statistical code, and dataset from which the results were derived.
