# Supplementary material for: Misclassification Bias in the Waist‐To‐Hip Ratio: Implications for Obesity‐Related Risk of Incident Type 2 Diabetes
Source: Int J Endocrinol. 2026 Jun 10;2026:1407398. doi: 10.1155/ije/1407398 (PMC13250689; doi:10.1155/ije/1407398)
Supplement: Supplementary file 1 — Supporting Information The supporting information file includes the flowchart of the study participant, supporting tables and figures of the main analysis, and the results of sensitivity analysis. [file IJE-2026-1407398-s001.docx]

**Flow chart**


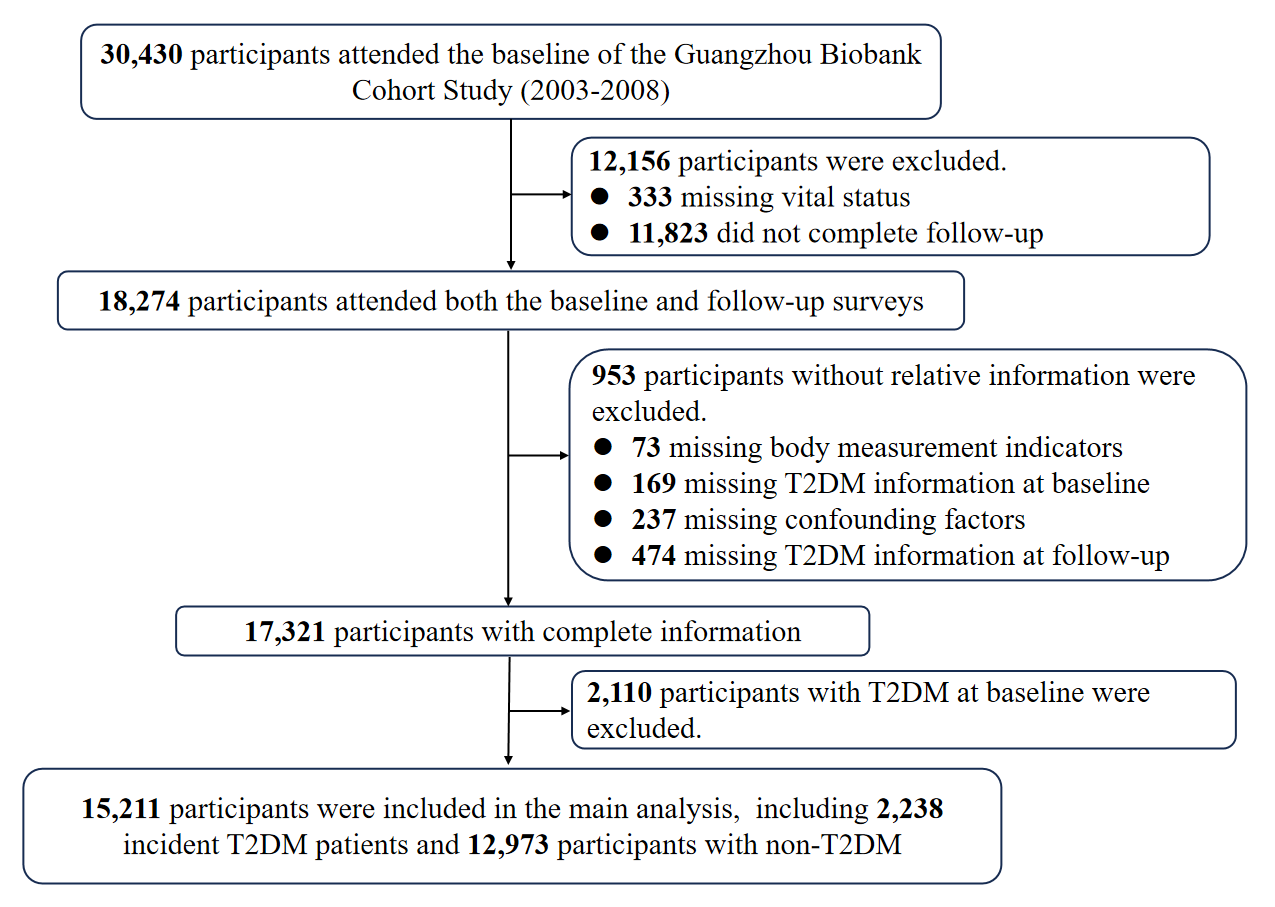


**Figure S1** Flow chart of the study participant

**Main analysis**

**Table S1** Associations between WC and HC with risk of incident T2DM across WHR stratifications

|  | Model 1 ^d^ | Model 2 ^e^ |
| --- | --- | --- |
| **women: WHR＜0.84;** **men: WHR＜0.90 ^a^** | | |
| WC |  |  |
| Small WC ^b^ | 1.00 (ref) | 1.00 (ref) |
| Large WC ^b^ | 2.21 (1.91, 2.57) ^***^ | 1.75 (1.41, 2.18) ^***^ |
| z-score (1 SD=8.36 cm in women; 8.86 cm in men) | 1.80 (1.64, 1.98) ^***^ | 2.80 (2.18, 3.61) ^***^ |
| HC |  |  |
| Small HC ^c^ | 1.00 (ref) | 1.00 (ref) |
| Large HC ^c^ | 1.77 (1.53, 2.06) ^***^ | 0.90 (0.73, 1.11) |
| z-score (1 SD=6.32 cm in women; 5.93 cm in men) | 1.40 (1.30, 1.50) ^***^ | 0.71 (0.59, 0.85) ^***^ |
| **women: WHR≥0.84; men: WHR≥0.90 ^a^** | | |
| WC |  |  |
| Small WC ^b^ | 1.00 (ref) | 1.00 (ref) |
| Large WC ^b^ | 1.63 (1.39, 1.91) ^***^ | 1.24 (1.03, 1.49) ^*^ |
| z-score (1 SD=8.36 cm in women; 8.86 cm in men) | 1.44 (1.35, 1.53) ^***^ | 1.54 (1.40, 1.71) ^***^ |
| HC |  |  |
| Small HC ^c^ | 1.00 (ref) | 1.00 (ref) |
| Large HC ^c^ | 1.48 (1.32, 1.65) ^***^ | 1.00 (0.87, 1.15) |
| z-score (1 SD=6.32 cm in women; 5.93 cm in men) | 1.24 (1.18, 1.30) ^***^ | 0.93 (0.86, 1.01) |

WHR=waist-to-hip ratio, WC=waist circumference, HC=hip circumference

^a^ WHR were divided by the sex-specific medians (women: 0.84; men: 0.90).

^b^ Small and large WC were divided by the sex-specific medians (women: 76 cm; men: 81 cm).

^c^ Small and large HC were divided by the sex-specific medians (women: 90 cm; men: 90 cm).

^d^ Model 1 adjusted for age, sex, education, occupation, family income, smoking status, alcohol use, physical activity.

^e^ Model 2 for estimating the associations between WC or HC with incident T2DM risk additionally adjusting for HC or WC.

^*^*P*<0.05, ^***^*P*<0.001


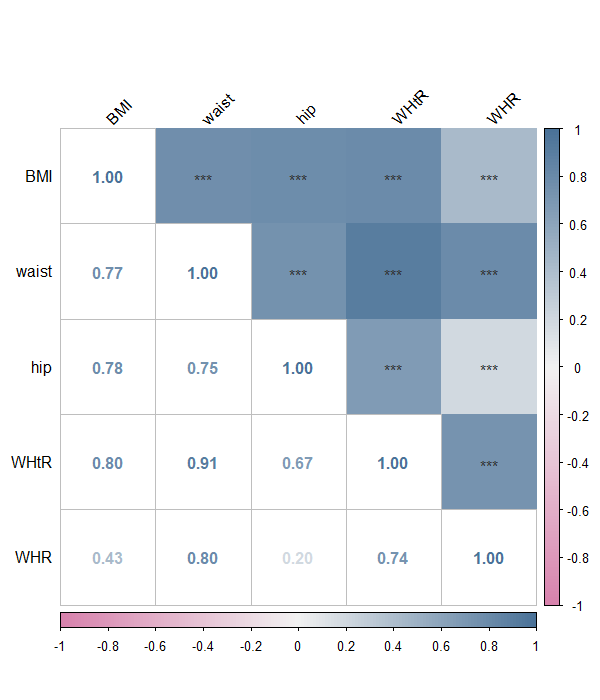


**Figure S2** Correlations among body measurement indicators and obesity indicators.

Note: The correlations among body measurement indicators and obesity indicators were evaluated using Pearson correlation coefficients (Pearson’s *r*).

^***^*P*<0.001

BMI=Body mass index, WHtR=waist-to-height ratio, WHR=waist-to-hip ratio


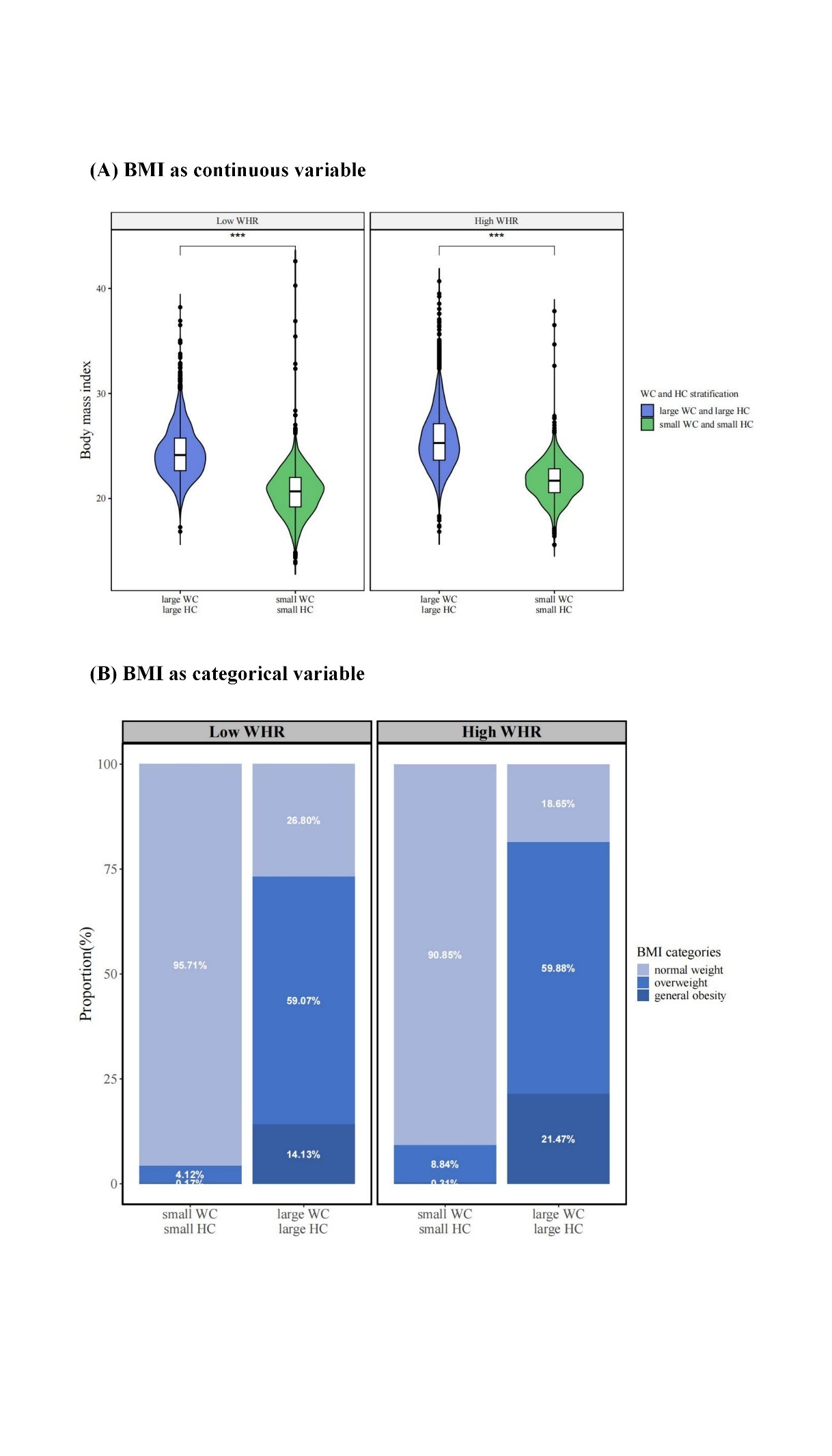


**Figure S3** The distribution of BMI among the category with small WC and small HC and that with large WC and large HC across WHR stratifications.

Note: Small and large WC were divided by the sex-specific medians (women: 76 cm; men: 81cm). Small and large HC were divided by the sex-specific medians (women: 90 cm; men: 90 cm). Low and high WHR were divided by the sex-specific medians (women: 0.84; men: 0.90). BMI was classified according to the Chinese criteria (normal weight: <24 kg/m^2^, overweight: 24 kg/m^2^≤BMI<28 kg/m^2^; general obesity: ≥28 kg/m^2^).

^***^*P*<0.001

T2DM=Type 2 diabetes, BMI=Body mass index, WHR=waist-to-hip ratio, WC=waist circumference, HC=hip circumference

**Table S2** Associations of WC and HC categories combined with BMI on incident T2DM risk across WHR stratifications

| Different categories of WHR, WC, HC and BMI | | | Newly developed T2DM/ Non-T2DM | Incidence rate,  per 1,000  person-years | HR  (95% CI) ^†^ |
| --- | --- | --- | --- | --- | --- |
| low WHR ^a^ | small WC and  small HC ^b^ | normal weight ^c^ | 271 /3613 | 11.42 | 1.00 (ref) |
|  |  | overweight ^c^ | 20 /147 | 20.72 | 1.75 (1.11, 2.76) ^*^ |
|  |  | obesity ^c^ | 0 /7 | - | - |
|  | large WC and  large HC ^b^ | normal weight | 68 /412 | 23.16 | 1.94 (1.48, 2.53) ^***^ |
|  |  | overweight | 176 /882 | 28.55 | 2.44 (2.02, 2.95) ^***^ |
|  |  | obesity | 52 /201 | 37.01 | 3.18 (2.36, 4.29) ^***^ |
| high WHR ^a^ | small WC and  small HC | normal weight ^c^ | 156 /1005 | 23.6 | 1.00 (ref) |
|  |  | overweight ^c^ | 15 /98 | 24.2 | 1.04 (0.61, 1.77) |
|  |  | obesity ^c^ | 0 /4 | - | - |
|  | large WC and  large HC | normal weight | 148 /759 | 28.64 | 1.21 (0.96, 1.51) |
|  |  | overweight | 592 /2320 | 37.73 | 1.58 (1.32, 1.89) ^***^ |
|  |  | obesity | 334 /710 | 65.64 | 2.68 (2.22, 3.24) ^***^ |

WHR=waist-to-hip ratio, WC=waist circumference, HC=hip circumference, BMI=Body mass index, T2DM=Type 2 diabetes, HR=hazard ratio, CI=confidence interval

^a^ Low and high WHR were divided by the sex-specific medians (women: 0.84; men: 0.90).

^b^ Small and large WC were divided by the sex-specific medians (women: 76 cm; men: 81cm). Small and large HC were divided by the sex-specific medians (women: 90 cm; men: 90 cm).

^c^ BMI was classified according to the Chinese criteria (normal weight: <24 kg/m^2^, overweight: 24 kg/m^2^≤BMI<28 kg/m^2^; general obesity: ≥28 kg/m^2^).

^†^ Model adjusted for age, sex, education, occupation, family income, smoking status, alcohol use, physical activity. The groups with small WC and HC combined with normal weight were considered as the reference groups.

^*^*P*<0.05, ^***^*P*<0.001

**Table S3** The joint associations of BMI and WHR on risk of incident T2DM

| Different categories of WHR and BMI | | Newly developed T2DM/ Non-T2DM | Incidence rate,  per 1,000  person-years | HR  (95% CI) ^†^ |
| --- | --- | --- | --- | --- |
| low WHR ^a^ | normal weight ^c^ | 430 /5101 | 12.68 | 1.00 (ref) |
|  | overweight ^c^ | 244 /1478 | 24.04 | 1.92 (1.64, 2.25) ^***^ |
|  | obesity ^c^ | 53 /217 | 35.25 | 2.80 (2.10, 3.72) ^***^ |
| high WHR ^a^ | normal weight ^c^ | 453 /2629 | 25.92 | 1.91 (1.67, 2.18) ^***^ |
|  | overweight ^c^ | 721 /2817 | 37.93 | 2.79 (2.47, 3.15) ^***^ |
|  | obesity ^c^ | 337 /731 | 64.67 | 4.63 (4.01, 5.35) ^***^ |

WHR=waist-to-hip ratio, WC=waist circumference, HC=hip circumference, BMI=Body mass index, T2DM=Type 2 diabetes, HR=hazard ratio, CI=confidence interval

^a^ Low and high WHR were divided by the sex-specific medians (women: 0.84; men: 0.90).

^b^ Small and large WC were divided by the sex-specific medians (women: 76 cm; men: 81cm). Small and large HC were divided by the sex-specific medians (women: 90 cm; men: 90 cm).

^c^ BMI was classified according to the Chinese criteria (normal weight: <24 kg/m^2^, overweight: 24 kg/m^2^≤BMI<28 kg/m^2^; general obesity: ≥28 kg/m^2^).

^†^ Model adjusted for age, sex, education, occupation, family income, smoking status, alcohol use, physical activity. The group with low WHR combined with normal weight was considered as the reference group.

^***^*P*<0.001

**Sensitivity analysis**

**Table S4** Comparisons of obesity indicators for associations with risk of incident T2DM and their discriminatory performance (stratified by sex)

|  | HR  (95% CI) ^†^ | C-statistic  (95% CI) | △C-statistic  (95% CI) | NRI  (95% CI) | IDI  (95% CI) |
| --- | --- | --- | --- | --- | --- |
| **Women** | | | | | |
| BMI (1 SD=3.26 kg/m^2^) | 1.55 (1.48, 1.62) ^***^ | 0.636 (0.623, 0.649) | 0.000 (-0.015, 0.014) | 0.007 (-0.041, 0.061) | 0.005 (-0.002, 0.013) |
| WC (1 SD=8.36 cm) | 1.62 (1.54, 1.70) ^***^ | 0.646 (0.633, 0.659) | 0.010 (0.000, 0.019) ^*^ | 0.088 (0.034, 0.128) ^***^ | 0.009 (0.004, 0.015) ^***^ |
| WHtR (1 SD=0.06) | 1.67 (1.59, 1.75) ^***^ | 0.654 (0.641, 0.667) | 0.017 (0.008, 0.027) ^***^ | 0.115 (0.060, 0.156) ^***^ | 0.014 (0.008, 0.019) ^***^ |
| WHR (1 SD=0.06) | 1.48 (1.42, 1.55) ^***^ | 0.636 (0.623, 0.649) | 0.000 (ref) | 0.000 (ref) | 0.000 (ref) |
| **Men** | | | | | |
| BMI (1 SD=3.10 kg/m^2^) | 1.55 (1.44, 1.67) ^***^ | 0.631 (0.609, 0.653) | -0.006 (-0.027, 0.016) | -0.030 (-0.109, 0.046) | -0.002 (-0.012, 0.008) |
| WC (1 SD=8.86 cm) | 1.66 (1.53, 1.79) ^***^ | 0.650 (0.629, 0.671) | 0.013 (-0.002, 0.028) | 0.039 (-0.044, 0.119) | 0.004 (-0.003, 0.012) |
| WHtR (1 SD=0.05) | 1.68 (1.55, 1.82) ^***^ | 0.655 (0.634, 0.676) | 0.018 (0.005, 0.032) ^**^ | 0.088 (-0.009, 0.159) | 0.007 (0.000, 0.014) |
| WHR (1 SD=0.06) | 1.58 (1.46, 1.71) ^***^ | 0.637 (0.616, 0.658) | 0.000 (ref) | 0.000 (ref) | 0.000 (ref) |

SD=standard deviation, CI=confidence interval, NRI=net reclassification index, IDI=integrated discrimination improvement, BMI=body mass index, WHtR=waist-to-height ratio, WHR=waist-to-hip ratio, WC=waist circumference, HC=hip circumference

**^†^** Adjusted for age, education, occupation, family income, smoking status, alcohol use, physical activity.

^*^*P*<0.05, ^**^*P*<0.01, ^***^*P*<0.001


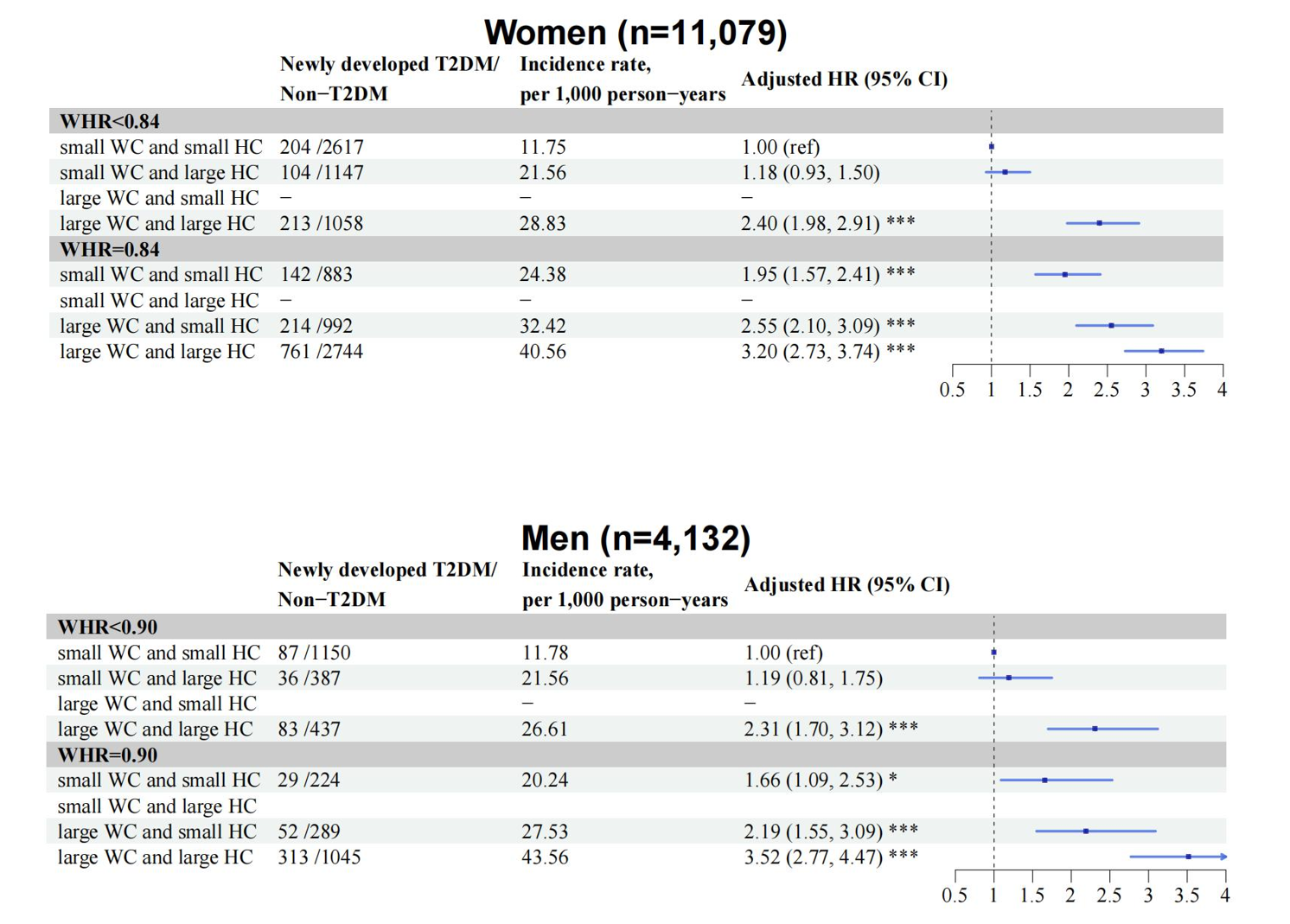


**Figure S4** Associations of WC and HC categories with risk of incident T2DM across WHR stratifications (stratified by sex).

Note: WHR were divided by the sex-specific medians (women: 0.84; men: 0.90). Small and large WC were divided by the sex-specific medians (women: 76 cm; men: 81cm). Small and large HC were divided by the sex-specific medians (women: 90 cm; men: 90 cm). Adjusted HRs and 95%CIs were adjusted for age, education, occupation, family income, smoking status, alcohol use, physical activity.

T2DM=Type 2 diabetes, WHR=waist-to-hip ratio, WC=waist circumference, HC=hip circumference

^**^*P*<0.01, ^***^*P*<0.001

**Table S5** Comparisons of discriminatory performance of combined BMI and central obesity indicators for predicting incident T2DM (stratified by sex)

|  | C-statistic  (95% CI) | △C-statistic  (95% CI) | NRI  (95% CI) | IDI  (95% CI) |
| --- | --- | --- | --- | --- |
| **Women** | | | | |
| BMI+WC | 0.649 (0.636, 0.662) | -0.011 (-0.015, -0.006) ^***^ | -0.115 (-0.159, -0.065) ^***^ | -0.004 (-0.007, -0.001) ^***^ |
| BMI+WHtR | 0.655 (0.642, 0.668) | -0.005 (-0.009, 0.000) ^*^ | -0.045 (-0.097, 0.013) | 0.000 (-0.004, 0.002) |
| BMI+WHR | 0.660 (0.647, 0.673) | 0.000 (ref) | 0.000 (ref) | 0.000 (ref) |
| WHR alone | 0.626 (0.615, 0.637) | -0.023 (-0.031, -0.015) ^***^ | -0.016 (-0.021, -0.011) ^***^ | -0.144 (-0.177, -0.114) ^***^ |
| **Men** | | | | |
| BMI+WC | 0.650 (0.629, 0.671) | -0.001 (-0.009, 0.008) | -0.075 (-0.142, -0.014) ^*^ | -0.003 (-0.008, 0.002) |
| BMI+WHtR | 0.655 (0.634, 0.676) | 0.004 (-0.004, 0.012) | -0.030 (-0.129, 0.051) | -0.001 (-0.006, 0.004) |
| BMI+WHR | 0.651 (0.630, 0.672) | 0.000 (ref) | 0.000 (ref) | 0.000 (ref) |
| WHR alone | 0.626 (0.615, 0.637) | -0.015 (-0.025, -0.004) ^*^ | -0.008 (-0.014, -0.003) ^***^ | -0.105 (-0.161, -0.047) ^***^ |

CI=confidence interval, NRI=net reclassification index, IDI=integrated discrimination improvement, BMI=body mass index, WC=waist circumference, WHtR=waist-to-height ratio, WHR=waist-to-hip ratio

^*^*P*<0.05, ^***^*P*<0.001

**Table S6** Associations of WC and HC categories combined with BMI on incident T2DM risk across WHR stratifications (stratified by sex)

| Different categories of WHR, WC, HC and BMI | | | Newly developed T2DM/ Non-T2DM | Incidence rate,  per 1,000  person-years | HR (95% CI) ^†^ |
| --- | --- | --- | --- | --- | --- |
| **Women** | | | | | |
| low WHR ^a^ | small WC and  small HC ^b^ | normal weight ^c^ | 189 /2511 | 11.35 | 1.00 (ref) |
|  |  | overweight ^c^ | 15 /103 | 22.14 | 1.94 (1.14, 3.29) ^*^ |
|  |  | obesity ^c^ | 0 /3 | - | - |
|  | large WC and  large HC ^b^ | normal weight | 35 /253 | 19.57 | 1.66 (1.16, 2.38) ^**^ |
|  |  | overweight | 138 /634 | 31.11 | 2.66 (2.14, 3.32) ^***^ |
|  |  | obesity | 40 /171 | 34.4 | 2.97 (2.11, 4.18) ^***^ |
| high WHR ^a^ | small WC and  small HC | normal weight ^c^ | 129 /796 | 24.49 | 1.00 (ref) |
|  |  | overweight ^c^ | 13 /84 | 24.16 | 1.00 (0.57, 1.78) |
|  |  | obesity ^c^ | 0 /3 | - | - |
|  | large WC and  large HC | normal weight | 97 /542 | 26.29 | 1.08 (0.83, 1.40) |
|  |  | overweight | 405 /1662 | 36.13 | 1.46 (1.19, 1.78) ^***^ |
|  |  | obesity | 259 /540 | 67.03 | 2.64 (2.13, 3.26) ^***^ |
| **Men** | | | | | |
| low WHR ^a^ | small WC and  small HC ^b^ | normal weight ^c^ | 82 /1102 | 11.58 | 1.00 (ref) |
|  |  | overweight ^c^ | 5 /44 | 17.39 | 1.32 (0.53, 3.27) |
|  |  | obesity ^c^ | 0 /4 | - | - |
|  | large WC and  large HC ^b^ | normal weight | 33 /159 | 28.75 | 2.39 (1.59, 3.60) ^***^ |
|  |  | overweight | 38 /248 | 21.98 | 1.92 (1.30, 2.84) ^**^ |
|  |  | obesity | 12 /30 | 49.57 | 4.55 (2.43, 8.53) ^***^ |
| high WHR ^a^ | small WC and  small HC | normal weight ^c^ | 27 /209 | 20.09 | 1.00 (ref) |
|  |  | overweight ^c^ | 2 /14 | 24.47 | 1.18 (0.28, 4.99) |
|  |  | obesity ^c^ | 0 /1 | - | - |
|  | large WC and  large HC | normal weight | 51 /217 | 34.5 | 1.69 (1.06, 2.70) ^*^ |
|  |  | overweight | 187 /658 | 41.71 | 2.10 (1.40, 3.15) ^***^ |
|  |  | obesity | 75 /170 | 61.24 | 3.12 (2.01, 4.86) ^***^ |

WHR=waist-to-hip ratio, WC=waist circumference, HC=hip circumference, BMI=Body mass index, T2DM=Type 2 diabetes, HR=hazard ratio, CI=confidence interval

^a^ Low and high WHR were divided by the sex-specific medians (women: 0.84; men: 0.90).

^b^ Small and large WC were divided by the sex-specific medians (women: 76 cm; men: 81cm). Small and large HC were divided by the sex-specific medians (women: 90 cm; men: 90 cm).

^c^ BMI was classified according to the Chinese criteria (normal weight: <24 kg/m^2^, overweight: 24 kg/m^2^≤BMI<28 kg/m^2^; general obesity: ≥28 kg/m^2^).

^†^ Model adjusted for age, education, occupation, family income, smoking status, alcohol use, physical activity. The groups with small WC and HC combined with normal weight were considered as the reference groups.

^*^*P*<0.05, ^**^*P*<0.01, ^***^*P*<0.001

**Table S7** The joint associations of BMI and WHR ratio with incident T2DM risk (stratified by sex)

| Different categories of WHR and BMI | | Newly developed T2DM/ Non-T2DM | Incidence rate,  per 1,000  person-years | HR (95% CI) ^†^ |
| --- | --- | --- | --- | --- |
| **Women** | | | | |
| low WHR ^a^ | normal weight ^c^ | 286 /3546 | 12.08 | 1.00 (ref) |
|  | overweight ^c^ | 194 /1093 | 25.88 | 2.13 (1.78, 2.56) ^***^ |
|  | obesity ^c^ | 41 /183 | 32.97 | 2.67 (1.93, 3.71) ^***^ |
| high WHR ^a^ | normal weight | 337 /2018 | 25.09 | 1.91 (1.63, 2.25) ^***^ |
|  | overweight | 519 /2041 | 37.67 | 2.87 (2.48, 3.33) ^***^ |
|  | obesity | 261 /560 | 65.61 | 4.90 (4.14, 5.81) ^***^ |
| **Men** | | | | |
| low WHR ^a^ | normal weight ^c^ | 144 /1555 | 14.09 | 1.00 (ref) |
|  | overweight ^c^ | 50 /385 | 18.83 | 1.38 (1.00, 1.91) |
|  | obesity ^c^ | 12 /34 | 46.15 | 3.50 (1.93, 6.34) ^***^ |
| high WHR ^a^ | normal weight | 116 /611 | 28.7 | 1.93 (1.51, 2.47) ^***^ |
|  | overweight | 202 /776 | 38.6 | 2.60 (2.10, 3.22) ^***^ |
|  | obesity | 76 /171 | 61.63 | 4.21 (3.18, 5.58) ^***^ |

WHR=waist-to-hip ratio, WC=waist circumference, HC=hip circumference, BMI=Body mass index, T2DM=Type 2 diabetes, HR=hazard ratio, CI=confidence interval

^a^ Low and high WHR were divided by the sex-specific medians (women: 0.84; men: 0.90).

^b^ Small and large WC were divided by the sex-specific medians (women: 76 cm; men: 81cm). Small and large HC were divided by the sex-specific medians (women: 90 cm; men: 90 cm).

^c^ BMI was classified according to the Chinese criteria (normal weight: <24 kg/m^2^, overweight: 24 kg/m^2^≤BMI<28 kg/m^2^; general obesity: ≥28 kg/m^2^).

^†^ Model adjusted for age, education, occupation, family income, smoking status, alcohol use, physical activity. The group with low WHR combined with normal weight was considered as the reference group.

^***^*P*<0.001

**Table S8** Associations of WC and HC categories with risk of incident T2DM across WHR stratifications (additionally adjusting for daily total energy intake)

|  | Newly developed T2DM/ Non-T2DM | Incidence rate,  per 1,000  person-years | HR  (95% CI) ^†^ |
| --- | --- | --- | --- |
| **women: WHR＜0.84; men: WHR＜0.90** ^a^ | | | |
| small WC and small HC ^b^ | 202 /2485 | 11.69 | 1.00 (ref) |
| small WC and large HC | 129 /1078 | 21.56 | 1.44 (1.15, 1.80) ^**^ |
| large WC and small HC | - | - | - |
| large WC and large HC | 186 /802 | 30.62 | 2.58 (2.11, 3.15) ^***^ |
| **women: WHR≥0.84; men: WHR≥0.90** ^a^ | | | |
| small WC and small HC ^b^ | 130 /842 | 21.93 | 1.80 (1.44, 2.25) ^***^ |
| small WC and large HC | 3 /32 | 14.41 | 1.21 (0.39, 3.79) |
| large WC and small HC | 173 /696 | 35.13 | 2.88 (2.34, 3.53) ^***^ |
| large WC and large HC | 749 /2434 | 41.93 | 3.38 (2.89, 3.95) ^***^ |

WHR=waist-to-hip ratio, WC=waist circumference, HC=hip circumference, HR=hazard ratio, CI=confidence interval

^a^ WHR were divided by the sex-specific medians (women: 0.85; men: 0.90).

^b^ Small and large WC were divided by the sex-specific medians (women: 77 cm; men: 82 cm). Small and large HC were divided by the sex-specific medians (women: 90 cm; men: 90.5 cm).

^†^ Model adjusted for age, education, occupation, family income, smoking status, alcohol use, physical activity and daily total energy intake.

^**^*P*<0.01, ^***^*P*<0.001

**Table S9** Associations of WC and HC categories combined with BMI on incident T2DM risk across WHR stratifications (additionally adjusting for daily total energy intake)

| Different categories of WHR, WC, HC and BMI | | | Newly developed T2DM/ Non-T2DM | Incidence rate,  per 1,000  person-years | HR (95% CI) ^†^ |
| --- | --- | --- | --- | --- | --- |
| low WHR ^a^ | small WC and  small HC ^b^ | normal weight c | 125/2251 | 12.61 | 1.00 (ref) |
|  |  | normal weight ^c^ | 188 /2390 | 11.3 | 1.00 (ref) |
|  |  | overweight ^c^ | 14 /90 | 22.66 | 1.93 (1.12, 3.33) ^*^ |
|  |  | obesity ^c^ | 0 /5 | - | - |
|  | large WC and  large HC ^b^ | normal weight | 39 /184 | 27.58 | 2.28 (1.61, 3.22) ^***^ |
|  |  | overweight | 112 /494 | 29.97 | 2.55 (2.01, 3.23) ^***^ |
|  |  | obesity | 35 /124 | 37.88 | 3.44 (2.39, 4.96) ^***^ |
| high WHR ^a^ | small WC and  small HC | normal weight ^c^ | 118 /771 | 21.65 | 1.00 (ref) |
|  |  | overweight ^c^ | 12 /71 | 25.1 | 1.14 (0.63, 2.07) |
|  |  | obesity ^c^ | - | - | - |
|  | large WC and  large HC | normal weight | 104 /493 | 29.05 | 1.34 (1.03, 1.74) ^*^ |
|  |  | overweight | 425 /1507 | 38.95 | 1.76 (1.44, 2.17) ^***^ |
|  |  | obesity | 220 /434 | 65.21 | 2.88 (2.30, 3.61) ^***^ |

WHR=waist-to-hip ratio, WC=waist circumference, HC=hip circumference, BMI=Body mass index, T2DM=Type 2 diabetes, HR=hazard ratio, CI=confidence interval

^a^ Low and high WHR were divided by the sex-specific medians (women: 0.84; male: 0.90).

^b^ Small and large WC were divided by the sex-specific medians (women: 76 cm; men: 81cm). Small and large HC were divided by the sex-specific medians (women: 90 cm; men: 90 cm).

^c^ BMI was classified according to the Chinese criteria (normal weight: <24 kg/m^2^, overweight: 24 kg/m^2^≤BMI<28 kg/m^2^; general obesity: ≥28 kg/m^2^).

^†^ Model adjusted for age, sex, education, occupation, family income, smoking status, alcohol use, physical activity and daily total energy intake. The groups with small WC and HC combined with normal weight were considered as the reference groups.

^*^*P*<0.05, ^***^*P*<0.001

**Table S10** The joint associations of BMI and WHR with incident T2DM risk (additionally adjusting for daily total energy intake)

| Different categories of WHR and BMI | | Newly developed T2DM/ Non-T2DM | Incidence rate,  per 1,000  person-years | HR (95% CI) ^†^ |
| --- | --- | --- | --- | --- |
| low WHR ^a^ | normal weight ^c^ | 305 /3312 | 13.01 | 1.00 (ref) |
|  | overweight ^c^ | 175 /918 | 25.86 | 2.00 (1.66, 2.41) ^***^ |
|  | obesity ^c^ | 37 /135 | 37.04 | 2.88 (2.04, 4.05) ^***^ |
| high WHR ^a^ | normal weight | 329 /1725 | 26.76 | 1.95 (1.67, 2.29) ^***^ |
|  | overweight | 505 /1838 | 38.21 | 2.76 (2.40, 3.19) ^***^ |
|  | obesity | 221 /441 | 64.69 | 4.57 (3.84, 5.44) ^***^ |

WHR=waist-to-hip ratio, WC=waist circumference, HC=hip circumference, BMI=Body mass index, T2DM=Type 2 diabetes, HR=hazard ratio, CI=confidence interval

^a^ Low and high WHR were divided by the sex-specific medians (women: 0.84; men: 0.90).

^b^ Small and large WC were divided by the sex-specific medians (women: 76 cm; men: 81cm). Small and large HC were divided by the sex-specific medians (women: 90 cm; men: 90 cm).

^c^ BMI was classified according to the Chinese criteria (normal weight: <24 kg/m^2^, overweight: 24 kg/m^2^≤BMI<28 kg/m^2^; general obesity: ≥28 kg/m^2^).

^†^ Model adjusted for age, sex, education, occupation, family income, smoking status, alcohol use, physical activity and daily total energy intake. The group with low WHR combined with normal weight was considered as the reference group.

^***^*P*<0.001

**Table S11** Associations of WC and HC categories with risk of incident T2DM across WHR stratifications (additionally adjusting for family history of diabetes)

|  | Newly developed T2DM/ Non-T2DM | Incidence rate,  per 1,000  person-years | HR (95% CI) ^†^ |
| --- | --- | --- | --- |
| **women: WHR＜0.84; men: WHR＜0.90** **^a^** | | | |
| small WC and small HC ^b^ | 134 /1261 | 13.23 | 1.00 (ref) |
| small WC and large HC | 71 /460 | 21.56 | 1.39 (1.04, 1.85) ^*^ |
| large WC and small HC | - | - | - |
| large WC and large HC | 100 /408 | 27.28 | 2.10 (1.62, 2.73) ^***^ |
| **women: WHR≥0.84; men: WHR≥0.90 ^a^** | | | |
| small WC and small HC ^b^ | 89 /450 | 25.85 | 1.78 (1.36, 2.34) ^***^ |
| small WC and large HC | - | - | - |
| large WC and small HC | 101 /429 | 30.48 | 2.18 (1.68, 2.83) ^***^ |
| large WC and large HC | 371 /1119 | 39.53 | 2.90 (2.37, 3.53) ^***^ |

WHR=waist-to-hip ratio, WC=waist circumference, HC=hip circumference, HR=hazard ratio, CI=confidence interval

^a^ WHR were divided by the sex-specific medians (women: 0.85; men: 0.90).

^b^ Small and large WC were divided by the sex-specific medians (women: 79 cm; men: 82.5 cm). Small and large HC were divided by the sex-specific medians (women: 92 cm; men: 92 cm).

^†^ Model adjusted for age, sex, education, occupation, family income, smoking status, alcohol use, physical activity and family history of diabetes.

^*^*P*<0.05, ^**^^*^*P*<0.001

**Table S12** Associations of WC and HC categories combined with BMI on incident T2DM risk across WHR stratifications (additionally adjusting for family history of diabetes)

| Different categories of WHR, WC, HC and BMI | | | Newly developed T2DM/ Non-T2DM | Incidence rate,  per 1,000  person-years | HR (95% CI) ^†^ |
| --- | --- | --- | --- | --- | --- |
| low WHR ^a^ | small WC and  small HC ^b^ | normal weight ^c^ | 124 /1211 | 12.86 | 1.00 (ref) |
|  |  | overweight ^c^ | 10 /47 | 38.02 | 2.17 (1.13, 4.15) ^*^ |
|  |  | obesity ^c^ | 0 /3 | - | - |
|  | large WC and  large HC ^b^ | normal weight | 15 /77 | 23.48 | 1.84 (1.07, 3.16) ^*^ |
|  |  | overweight | 61 /259 | 27.32 | 2.09 (1.53, 2.85) ^***^ |
|  |  | obesity | 24 /72 | 40.57 | 2.94 (1.88, 4.60) ^***^ |
| high WHR ^a^ | small WC and  small HC | normal weight | 78 /408 | 25.5 | 1.00 (ref) |
|  |  | overweight | 11 /42 | 33.72 | 1.35 (0.72, 2.55) |
|  |  | obesity | - | - | - |
|  | large WC and  large HC | normal weight | 47 /176 | 27.67 | 1.38 (0.96, 1.98) |
|  |  | overweight | 208 /717 | 37.62 | 1.46 (1.12, 1.90) ^**^ |
|  |  | obesity | 116 /226 | 62.83 | 2.38 (1.78, 3.18) ^***^ |

WHR=waist-to-hip ratio, WC=waist circumference, HC=hip circumference, BMI=Body mass index, T2DM=Type 2 diabetes, HR=hazard ratio, CI=confidence interval

^a^ Low and high WHR were divided by the sex-specific medians (women: 0.84; men: 0.90).

^b^ Small and large WC were divided by the sex-specific medians (women: 76 cm; men: 81cm). Small and large HC were divided by the sex-specific medians (women: 90 cm; men: 90 cm).

^c^ BMI was classified according to the Chinese criteria (normal weight: <24 kg/m^2^, overweight: 24 kg/m^2^≤BMI<28 kg/m^2^; general obesity: ≥28 kg/m^2^).

^†^ Model adjusted for age, sex, education, occupation, family income, smoking status, alcohol use, physical activity and family history of diabetes. The groups with small WC and HC combined with normal weight were considered as the reference groups.

^*^*P*<0.05, ^**^*P*<0.01, ^***^*P*<0.001

**Table S13** The joint associations of BMI and WHR with incident T2DM risk (additionally adjusting for family history of diabetes)

| Different categories of WHR and BMI | | Newly developed T2DM/ Non-T2DM | Incidence rate,  per 1,000  person-years | HR (95% CI) ^†^ |
| --- | --- | --- | --- | --- |
| low WHR ^a^ | normal weight ^c^ | 187 /1584 | 15.1 | 1.00 (ref) |
|  | overweight ^c^ | 93 /467 | 24.25 | 1.62 (1.26, 2.08) ^***^ |
|  | obesity ^c^ | 25 /78 | 38.88 | 2.45 (1.61, 3.73) ^***^ |
| high WHR ^a^ | normal weight | 179 /857 | 27.36 | 1.76 (1.43, 2.16) ^***^ |
|  | overweight | 264 /912 | 37.72 | 2.34 (1.94, 2.83) ^***^ |
|  | obesity | 118 /229 | 62.37 | 3.85 (3.05, 4.87) ^***^ |

WHR=waist-to-hip ratio, WC=waist circumference, HC=hip circumference, BMI=Body mass index, T2DM=Type 2 diabetes, HR=hazard ratio, CI=confidence interval

^a^ Low and high WHR were divided by the sex-specific medians (women: 0.84; men: 0.90).

^b^ Small and large WC were divided by the sex-specific medians (women: 76 cm; men: 81cm). Small and large HC were divided by the sex-specific medians (women: 90 cm; men: 90 cm).

^c^ BMI was classified according to the Chinese criteria (normal weight: <24 kg/m^2^, overweight: 24 kg/m^2^≤BMI<28 kg/m^2^; general obesity: ≥28 kg/m^2^).

^†^ Model adjusted for age, sex, education, occupation, family income, smoking status, alcohol use, physical activity and family history of diabetes. The group with low WHR combined with normal weight was considered as the reference group.

^***^*P*<0.001

**Table S14** Associations of WC and HC categories with risk of incident T2DM across WHR stratifications (according to WHO criteria)

|  | Newly developed T2DM/ Non-T2DM | Incidence rate,  per 1,000  person-years | HR (95% CI) ^†^ |
| --- | --- | --- | --- |
| **women: WHR＜0.85; men: WHR＜0.90 ^a^** | | | |
| small WC and small HC ^b^ | 326 /4028 | 12.3 | 1.00 (ref) |
| small WC and large HC | 354 /2758 | 21.56 | 1.55 (1.33, 1.80) ^***^ |
| large WC and small HC | - | - | - |
| large WC and large HC | 136 /618 | 31.44 | 2.49 (2.04, 3.05) ^***^ |
| **women: WHR≥0.85; men: WHR≥0.90 ^a^** | | | |
| small WC and small HC ^b^ | 299 /1747 | 26 | 2.00 (1.71, 2.34) ^***^ |
| small WC and large HC | - | - | - |
| large WC and small HC | 103 /380 | 41.5 | 3.05 (2.43, 3.82) ^***^ |
| large WC and large HC | 833 /2668 | 45.37 | 3.41 (2.99, 3.88) ^***^ |

WHR=waist-to-hip ratio, WC=waist circumference, HC=hip circumference, HR=hazard ratio, CI=confidence interval

^a^ WHR were divided by the WHO criteria (women: 0.85; men: 0.90).

^b^ Small and large WC were divided by the WHO criteria (women: 80 cm; men: 90 cm). Small and large HC were divided by the sex-specific medians (women: 92 cm; men: 92 cm).

^†^ Model adjusted for age, sex, education, occupation, family income, smoking status, alcohol use, and physical activity.

^***^*P*<0.001

**Table S15** Associations of WC and HC categories combined with BMI on incident T2DM risk across WHR stratifications (according to WHO criteria)

| Different categories of WHR, WC, HC and BMI | | | Newly developed T2DM/ Non-T2DM | Incidence rate,  per 1,000  person-years | HR (95% CI) ^†^ |
| --- | --- | --- | --- | --- | --- |
| low WHR ^a^ | small WC and  small HC ^b^ | normal weight ^c^ | 302 /3845 | 11.93 | 1.00 (ref) |
|  |  | overweight ^c^ | 24 /176 | 20.66 | 1.69 (1.11, 2.56) ^*^ |
|  |  | obesity ^c^ | 0 /7 | - | - |
|  | large WC and  large HC ^b^ | normal weight | 9 /44 | 28.6 | 2.15 (1.11, 4.19) ^*^ |
|  |  | overweight | 76 /383 | 28.53 | 2.24 (1.73, 2.89) ^***^ |
|  |  | obesity | 51 /191 | 37.85 | 3.04 (2.25, 4.10) ^***^ |
| high WHR ^a^ | small WC and  small HC | normal weight | 234 /1431 | 24.9 | 1.00 (ref) |
|  |  | overweight | 65 /311 | 31.38 | 1.29 (0.98, 1.71) |
|  |  | obesity | 0 /5 | - | - |
|  | large WC and  large HC | normal weight | 69 /354 | 28.45 | 1.16 (0.89, 1.52) |
|  |  | overweight | 450 /1661 | 40.04 | 1.59 (1.36, 1.86) ^***^ |
|  |  | obesity | 314 /653 | 66.88 | 2.62 (2.21, 3.10) ^***^ |

WHR=waist-to-hip ratio, WC=waist circumference, HC=hip circumference, BMI=Body mass index, T2DM=Type 2 diabetes, HR=hazard ratio, CI=confidence interval

^a^ Low and high WHR were divided by the WHO criteria (women: 0.85; men: 0.90).

^b^ Small and large WC were divided by the WHO criteria (women: 80 cm; men: 90 cm). Small and large HC were divided by the sex-specific medians (women: 90 cm; men: 90 cm).

^c^ BMI was classified according to the Chinese criteria (normal weight: <24 kg/m^2^, overweight: 24 kg/m^2^≤BMI<28 kg/m^2^; general obesity: ≥28 kg/m^2^).

^†^ Model adjusted for age, sex, education, occupation, family income, smoking status, alcohol use, and physical activity. The groups with small WC and HC combined with normal weight were considered as the reference groups.

^*^*P*<0.05, ^***^*P*<0.001

**Table S16** The joint associations of BMI and WHR with incident T2DM risk (according to WHO criteria)

| Different categories of WHR and BMI | | Newly developed T2DM/ Non-T2DM | Incidence rate,  per 1,000  person-years | HR (95% CI) ^†^ |
| --- | --- | --- | --- | --- |
| low WHR ^a^ | normal weight ^c^ | 469 /5426 | 13 | 1.00 (ref) |
|  | overweight ^c^ | 280 /1724 | 23.82 | 1.84 (1.59, 2.14) ^***^ |
|  | obesity ^c^ | 67 /254 | 37.45 | 2.86 (2.21, 3.69) ^***^ |
| high WHR ^a^ | normal weight | 414 /2304 | 27.06 | 1.93 (1.69, 2.21) ^***^ |
|  | overweight | 685 /2571 | 39.35 | 2.81 (2.50, 3.17) ^***^ |
|  | obesity | 323 /694 | 65.58 | 4.57 (3.96, 5.27) ^***^ |

WHR=waist-to-hip ratio, WC=waist circumference, HC=hip circumference, BMI=Body mass index, T2DM=Type 2 diabetes, HR=hazard ratio, CI=confidence interval

^a^ Low and high WHR were divided by the WHO criteria (women: 0.85; men: 0.90).

^b^ Small and large WC were divided by the WHO criteria (women: 80 cm; men: 90 cm). Small and large HC were divided by the sex-specific medians (women: 90 cm; men: 90 cm).

^c^ BMI was classified according to the Chinese criteria (normal weight: <24 kg/m^2^, overweight: 24 kg/m^2^≤BMI<28 kg/m^2^; general obesity: ≥28 kg/m^2^).

^†^ Model adjusted for age, sex, education, occupation, family income, smoking status, alcohol use, and physical activity. The group with low WHR combined with normal weight was considered as the reference group.

^***^*P*<0.001
